# Relationship Between Internet Health Information and Patient Compliance Based on Trust: Empirical Study

**DOI:** 10.2196/jmir.9364

**Published:** 2018-08-17

**Authors:** Xinyi Lu, Runtong Zhang, Wen Wu, Xiaopu Shang, Manlu Liu

**Affiliations:** ^1^ School of Economics and Management Beijing Jiaotong University Beijing China; ^2^ Saunders College of Business Rochester Institute of Technology Rochester, NY United States

**Keywords:** affect-based trust, cognition-based trust, internet health information, patient compliance, patient-physician relationship, social information processing theory, social exchange theory, structural equation modeling

## Abstract

**Background:**

The internet has become a major mean for acquiring health information; however, Web-based health information is of mixed quality and may markedly affect patients’ health-related behavior and decisions. According to the social information processing theory, patients’ trust in their physicians may potentially change due to patients’ health-information-seeking behavior. Therefore, it is important to identify the relationship between internet health information and patient compliance from the perspective of trust.

**Objective:**

The objective of our study was to investigate the effects of the quality and source of internet health information on patient compliance using an empirical study based on the social information processing theory and social exchange theory.

**Methods:**

A Web-based survey involving 336 valid participants was conducted in China. The study included independent variables (internet health information quality and source of information), 2 mediators (cognition-based trust [CBT] and affect-based trust [ABT]), 1 dependent variable (patient compliance), and 3 control variables (gender, age, and job). All variables were measured using multiple-item scales from previously validated instruments, and confirmative factor analysis as well as structural equation modeling was used to test hypotheses.

**Results:**

The questionnaire response rate was 77.16% (375/486), validity rate was 89.6% (336/375), and reliability and validity were acceptable. We found that the quality and source of internet health information affect patient compliance through the mediation of CBT and ABT. In addition, internet health information quality has a stronger influence on patient compliance than the source of information. However, CBT does not have any direct effect on patient compliance, but it directly affects ABT and then indirectly impacts patient compliance. Therefore, the effect of ABT seems stronger than that of CBT. We found an unexpected, nonsignificant relationship between the source of internet health information and ABT.

**Conclusions:**

From patients’ perspective, internet health information quality plays a stronger role than its source in impacting their trust in physicians and the consequent compliance with physicians. Therefore, patient compliance can be improved by strengthening the management of internet health information quality. The study findings also suggest that physicians should focus on obtaining health information from health websites, thereby expanding their understanding of patients’ Web-based health-information-seeking preferences, and enriching their knowledge structure to show their specialization and reliability in the communication with patients. In addition, the mutual demonstration of care and respect in the communication between physicians and patients is important in promoting patients’ ABT in their physicians.

## Introduction

### Background

The patient-physician relationship has become the second most important relationship, following the family relationship [1]. Communication plays an important role in the patient-physician relationship, and patient compliance is important in patient-physician interactions and receives considerable attention from scholars. Specifically, medical diagnoses and treatments can be effective if patients follow their physicians’ directions [2]. Patients’ attitudes and self-management are proposed to be critical in preventing diseases and promoting communication with physicians [3]. Therefore, the treatment effects of highly compliant patients are better than that of patients with low compliance [4].

Traditionally, patients have been primarily obtaining health information from physicians [5]. However, today, patients have begun to take advantage of the wide range of health information sources available beyond their physicians, including family, friends, and traditional mass media [6]. However, information from these sources cannot meet patients’ increasing demands [7]; moreover, patients are sensitive [8] regarding whether they would like to seek information on their own in order to monitor, for example, their physicians’ decision making. Also, patients with minor symptoms may prefer to diagnose themselves [9]. The development of the internet has provided patients with a considerable amount of health information [10], and thus, the internet has almost become a major source for patients to seek health information [11]. Nonetheless, health information available on the internet is of mixed quality, and some information is oversimplified, incomplete, inaccurate, or even misleading [12]. The current health information environment provides patients with an access to the information of different quality, and the information can directly influence patients’ trust in physicians and their decisions to follow physicians’ advice [13]. For example, if the health information from the internet is inconsistent with that from physicians, patients may doubt the advice of their physicians.

The relationship between internet health information and patient compliance has been studied on the basis of a perceived information asymmetry for patients [1]. This study, however, actually uses the perspective of psychology to study patient compliance. Reportedly, the interrelationship between patients’ behavior and their health is complex, and psychological factors, such as motivation, play an important role in this relationship [14]. Recently, researchers have begun to focus on behavioral psychology as well as its application in healthy choices and patient-physician interactions [15]. However, related previous studies that have discussed patient compliance from the angle of psychology are limited. Patient compliance, in fact, is a dynamic parameter that sometimes changes unintentionally because of cognitive deficiencies such as poor attention. In addition, noncompliance stems from other factors such as psychosocial stress [16]. Therefore, in this study, based on the social processing theory and social exchange theory, we intended to explore how internet health information impacts patient compliance through mediation of trust.

### Internet Health Information

The internet has become an important source of health information [17]. An increasing number of institutions, including governments, medical institutions, and business corporations, have established health information portals to provide public health information and to meet growing demands for such information [18]. However, problems like confusion and uncertainty about information quality remain serious. For example, mismatches between the Web-based health information obtained by patients and the actual demands of patients may arise [19]. Hence, it is important to conduct studies focusing on the internet health information. Previous studies have proposed that many people worry about the quality of internet health information, which is an important problem [20]. A marked and indirect relationship between the quality of internet health information and patient compliance has been identified; however, information asymmetry is considered to be a nonsignificant mediator [1]. Therefore, in this study, we used trust as another mediator between internet health information quality and patient compliance. Internet health information quality is used [1] to describe the information fitness for use and information reliability [3], comprising 4 dimensions: relevance, understandability, adequacy, and usefulness [1]. In terms of quantity, patients commonly acquire health information through search engines, such as Google, Bing, and Yahoo, and many health websites are available [12]; thus, a majority of patients have access to a great amount of internet health information regardless of their health literacy. Moreover, they tend to trust and select the first few results provided by these search engines [21]. On the other hand, quality includes adequacy as a dimension. In that case, we did not consider the quantity of Web-based health information in this study.

The source of internet health information is a critical attribute [22], and its evaluation in the health-information-seeking process is important [23]. Numerous health websites exist that have been built by several institutions, and one factor influencing patients’ selection of websites is their trust [6]. Therefore, how the source of internet health information impacts patient compliance should also be considered. The source of internet health information can be described using several related attributes (eg, reliability, accessibility, trustworthiness, and authority) [21,24]. In this study, we have described the source of internet health information with reliability, authority, and accessibility. In summary, we have discussed internet health information from the perspectives of its quality and source.

### Patient Compliance

Patient compliance is an important term that represents how patients follow the medical diagnoses and treatment regimens recommended by their physicians [1]; it plays a vital role in the patient-physician relationship. Patients’ attitudes and personal involvement, including self-management and self-monitoring, are all propitious to the improvement of patient compliance [8]. Patient compliance is mainly manifested in 2 aspects as follows: (1) maintaining a healthy lifestyle by following physicians’ advice and (2) medicine adherence. Khera et al [25], Stonerock and Blumenthal [26], and Johal et al [27] proposed that maintaining a healthy lifestyle is beneficial for preventing cardiovascular disease and reducing its incidence. In addition, living a healthy life has a substantial effect on decreasing the risk of cancer [28,29]. Nevertheless, when physicians suggest that patients make major changes in lifestyle, the patients are unlikely to comply [26]. In terms of medicine, for example, Varleta et al [30] concluded that a lack of adherence to taking medicines according to the prescription may lead to poor blood pressure control.

Noncompliance in health care may have three consequences. (1) For patients: the probability of morbidity and mortality is likely to increase [31]. (2) For economy: medical productivity and resources may be wasted because patients ignore the medical diagnoses and treatment regimens recommended by their physicians [31,32]. (3) For society: the use of genuinely beneficial drugs may be terminated due to noncompliance in clinical practice [31]. The treatment of chronic diseases largely relies on self-management and self-monitoring by patients [4]; thus, improving patient compliance may lead to better health-related outcomes than discovering any new therapy, given the increasing proportion of patients with chronic diseases [32]. However, the proportion of high compliant patients is relatively low, with a study revealing the rate of patients’ noncompliance with medicine to be as high as 50% [32]. Therefore, in this study, we aimed to discuss the improvement of patient compliance.

### Trust

Patients’ trust in their physicians is the core of the patient-physician relationship [33]. Trust has been discussed and defined in many previous studies, and this study focuses on the interpersonal trust between patients and physicians. Interpersonal trust is a pervasive phenomenon defined as “the extent to which a person is confident in, and willing to act on the basis of, the words, actions, and decisions of another” [34]. Furthermore, interpersonal trust is conceptualized into two different dimensions [34]: (1) cognition-based trust (CBT), which is grounded in the available knowledge, competence, and responsibility of individuals [35], and (2) affect-based trust (ABT), which is grounded in mutual respect, genuine care, and concern for the needs of others [36]. The patient-physician relationship is an important interpersonal relationship. The trust that patients have in physicians has been studied frequently; however, previous literature has rarely studied CBT and ABT in the patient-physician relationship. Nonetheless, these two types of trust have been considered to be important factors in behavioral studies [37], and they explain behavior from different points. Specifically, CBT is associated with individuals’ perceived competence and ABT with emotional connections [38]. Furthermore, cognition and affect are strongly linked [39], and CBT is built more easily than ABT [37]. Therefore, to effectively study the patient-physician relationship, trust is considered on both the cognitive and affective levels [40].

In CBT, when patients perceive their physicians as reliable, competent, and likely to offer useful help, they may be willing to trust these physicians [41]. While exchanging health information with their physicians, if the patients find that their physicians have shared or are sharing health information that is consistent with what they obtain from the internet, they may presume that their physicians are professional; thus, they will cognitively trust these physicians [41]. Consequently, they may follow the medical diagnoses and treatment regimens that these physicians propose. Regarding ABT, when an emotional connection is established, patients may feel safer while communicating with their physicians. Thus, they may be willing to show their vulnerability and are likely to express their personal attitudes related to health [41]. Therefore, disagreements, conflicts, and biased speculations can be reduced because of the full processing of information between patients and physicians [41]. In addition, CBT can impact ABT [34].

### Model and Hypotheses

**Figure 1:**
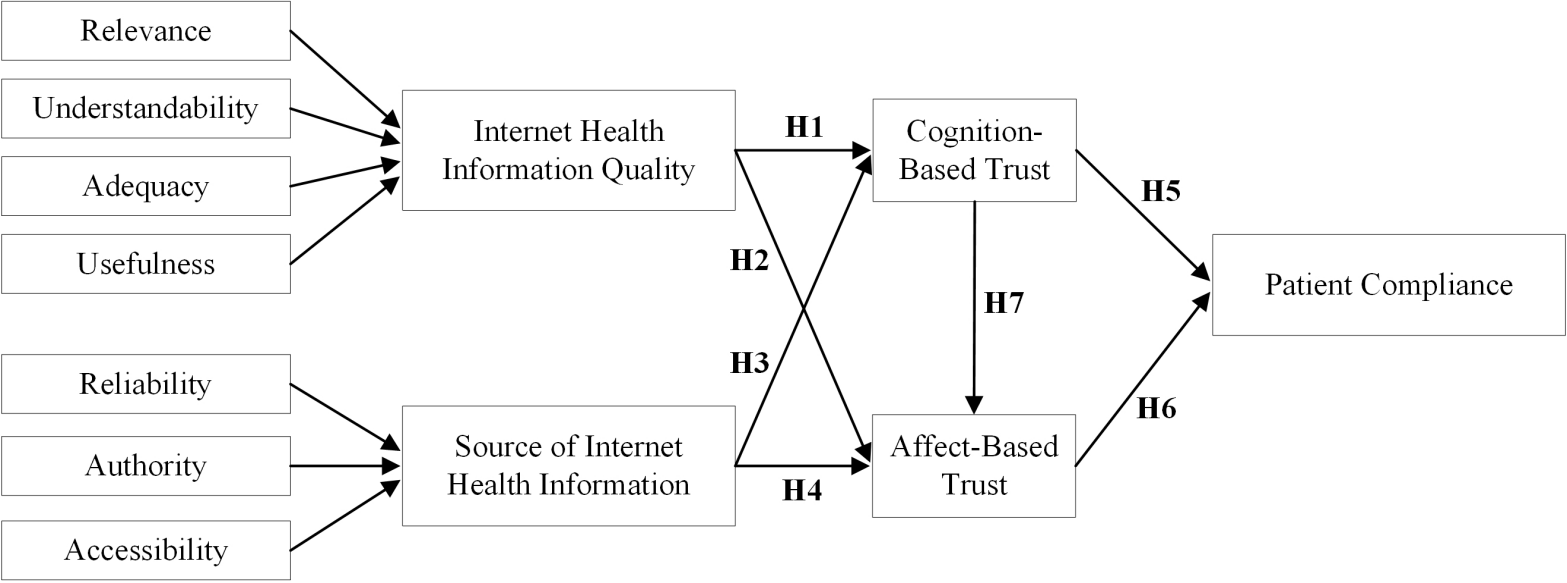
Research model.

Internet health information is advantageous to health-related decision making and patient-physician communication [42]. In this study, we investigated patient compliance by focusing on internet health information. Figure 1 shows the research model describing how internet health information impacts patient compliance through the mediation of CBT and ABT.

According to the social information processing theory [13], social contexts provide environmental cues, such as social information, which influence people’s behavioral options [43]. Especially when individuals do not have sufficient suitable information related to their targets, they are more likely to seek information from other sources, which may shape their attitudes, beliefs, and opinions [44]. In this study, we applied the social information processing theory to the patient-physician relationship, in the context of internet health information. Before or after visiting physicians, patients tend to seek health information from other sources in addition to physicians, such as books, news, mass media, and, especially, the internet [1]. Patients compare this obtained health information with that obtained from the patient-physician communication, which further helps establish their attitudes toward their physicians and may, in turn, influence their treatment behavior [45], such as patient compliance. In addition, the patient-physician relationship is a type of social exchange relationship [46], in which resources (love, status, information, money, goods, and services) are exchanged between physicians and patients [47,48]. Patients want to acquire correct information and suitable treatment, and physicians want to achieve the satisfaction of their patients, which eventually enhances doctors’ reputations. In that case, patients’ attitudes based on social information help them decide whether or not to exchange resources with their physicians. For example, if patients hold the view that their physician is unprofessional, they may switch to another physician and even provide negative comments about the first physician when other patients ask their advice.

To date, the significant relationship between internet health information quality and patients’ trust in physicians has not been directly supported by evidence. Reportedly, trust is a dynamic parameter [49], and the trust that patients have in their physicians may change because of certain influencing factors [50]. When patients obtain high-quality health information from the internet, they are likely to establish correct health-related views and beliefs, which, in turn, may cause them to realize that their physicians have truly shared useful health-related knowledge with them [1]. In such a case, patients are willing to trust their physicians. Hence, we proposed the following hypotheses:

H1: Internet health information quality has a positive impact on patients’ CBT in their physicians.

H2: Internet health information quality has a positive impact on patients’ ABT in their physicians.

Undoubtedly, the trust that patients place in internet health information changes with the information source, and the use of such information is influenced by their trust [6]. A good information source, such as an official health website, can provide high-quality information and help patients correctly understand their health conditions, as well as their physicians’ medical diagnoses and treatment regimens. Therefore, patients may perceive that they have received or are receiving suitable therapies from their physicians, which promotes patients’ trust in their physicians. Therefore, we proposed the following hypotheses:

H3: The source of internet health information has a positive impact on patients’ CBT in their physicians.

H4: The source of internet health information has a positive impact on patients’ ABT in their physicians.

As an interpersonal relationship, interpersonal trust plays an important role in the physician-patient relationship [50,51] and is also one of the critical principles of effective social exchange [46]. A previous study has proposed that CBT and ABT have direct effects on behaviors [52]. Regarding CBT, when the Web-based health information is consistent with that obtained from the physicians, the patients may cognitively trust their physicians. Then, the reliability and competence of physicians could be established from the patients’ points of view [34], by removing the uncertainty of the patient-physician relationship [53]. Consequently, patients are increasingly willing to trust the information provided by their physicians [41], and they feel the obligation to comply with physicians’ medical diagnoses and treatment regimens [54]. Therefore, we proposed the following hypothesis:

H5: Patients’ CBT in their physicians has a positive impact on the compliance.

In the patient-physician relationship, ABT is conducive to establishing an emotional connection based on mutual respect, care, and concern [36] and promoting a sense of safety in patients about expressing themselves when they interact with their physicians. Based on the social information processing theory, patients’ ABT in their physicians comes from the realization that their physicians are reliable and dependable, as evidenced by the health information that the patients have obtained through the internet. Consequently, patients tend to feel that they are taken seriously and are, thus, willing to communicate with their physicians and obey their physicians’ recommendations, as the reciprocation of physicians’ sincere treatments. Thus, disagreements, conflicts, and biased speculations may be reduced [41], and patients’ trust may lead to a high patient compliance. Consequently, we suggested the following hypothesis:

H6: Patients’ ABT in their physicians has a positive impact on the compliance.

CBT impacts ABT [34] and promotes patients to feel at ease in response to a reliable and professional atmosphere. In turn, patients come to regard their physicians as being reliable and gradually become emotionally dependent on them; consequently, ABT is established. Hence, this situation led us to derive the following hypothesis:

H7: Patients’ CBT in their physicians has a positive impact on their ABT in physicians.

## Methods

### Instrument Development

We used a multiple-item measurement scale to measure the constructs and previously validated instruments, which have been used in published works, for instrument development to ensure reliability and validity. A 7-point Likert-type response format that ranged from “strongly disagree” to “strongly agree” was used to measure items. The 5 variables in the research model (see Figure 1) were covered by the survey instrument (Multimedia Appendix 1). Patient compliance, which was discussed from a different perspective by Laugesen et al [1], was measured using a 5-item scale from the same reference. CBT and ABT were proposed by Mcallister [34] and measured by the same author using 6-item and 5-item scales, respectively. To address the subject of this study, these two scales were adjusted for measuring the CBT and ABT of patients in physicians. Internet health information quality consisted of the factors of relevance, understandability, adequacy, and usefulness [1] and was measured using a 16-item scale from Laugesen et al [1]. Similarly, the source of the internet health information, comprising the dimensions of reliability, authority, and accessibility, was measured using a 28-item scale divided into 3 parts. Specifically, reliability was measured using a scale from Singh et al [55] and authority and accessibility measured using an 18-item and a 5-item scale from Provost et al [24], respectively.

### Analysis Tool Selection

Research methods from previous studies [1,56] have used structural equation modeling (SEM) to analyze relationships between variables and to test hypotheses. In contrast with simple correlation-based models, we included mediators and complex relationships between variables in this research model. SEM has been widely accepted in several studies [57], and it accommodates intricate causal networks [1,58], such as testing hypotheses covering all variables and analyzing causal relationships in research models [59]. We used SEM for the following two reasons: (1) the measurement error can be incorporated, and the detected effects can be provided power through SEM and (2) the research model can be improved through a combination with confirmative factor analysis [60]. We used IBM’s SPSS 22.0 and Amos 22.0 (Armonk, New York, United States), which can achieve efficient and unbiased analysis and evaluate the latent variable interactions.

### Data Collection and Respondent Profile

The scales had to be translated into Chinese because the questionnaires would be distributed among Chinese respondents in China. First, as was done with the translation process in previous works [61,62], we recruited native Chinese speakers who had a master’s degree and above and were fluent in speaking English as well as skilled in scientific research translation to translate our scales into Chinese. As cross-cultural adaptation had to be considered [63], certain expressions needed to be modified. Second, 10 individuals from different professions and different ages, genders, and educational levels were invited to read the translated scales and provide recommendations for our modifying scales, consequently ensuring the comprehensibility, appropriateness, and readability in the context of Chinese culture. Finally, the scales underwent a reverse translation process performed by an English-speaking professional to check for the conceptual discrepancies and to ensure consistency with the original English version.

In the weeks preceding the formal investigation, a pretest was conducted with 112 subjects to ensure the clarity, conciseness, and readability of the scales and to determine the approximate time required to complete the questionnaire. Our subjects were Chinese individuals who had received medical therapies within the previous month and had sought Web-based health information. The formal investigation was anonymously conducted through a Web-based questionnaire survey addressed to participants in June 2017. The respondents were assured that their privacy was protected, and their informed consent was secured. Moreover, to control the duplication of responses, we only accepted the first response from the same IP address and deleted other responses within 1 hour. Of course, we did not tell participants this rule.

With the help of a medical association in China, we sent questionnaires to 486 participants and received a total of 375 responses, 336 of which were valid and covered 28 provinces of China (except Macao, Qinghai, Ningxia, Tibet, Xinjiang, and Hainan). Therefore, the response rate was 77.16% (375/486) and the validity rate was 89.6% (336/375). Table 1 presents the demographics of the research sample, in which 56.5% (190/336) participants were 20-40 years old, 53.6% (180/336) were females, and 62.2% (209/336) had, at least, a college education or a bachelor’s degree. Thus, more than half of this sample was young, female, and highly educated. A previous study has also reported that internet health information users are likely to be young, female, and educated [64]. The objective of our study was to identify the relationship between internet health information and patient compliance, and the investigative channel used was the internet. Therefore, the sample met our requirements.

**Table 1:** Sample demographics (N=336).

Demographic characteristics		Participants, n (%)
**Age (years)**		
	<20	22 (6.6%)
	20-29	83 (24.7%)
	30-39	107 (31.8%)
	40-49	59 (17.6%)
	50-59	47 (14.0%)
	≥60	18 (5.4%)
**Gender**		
	Male	156 (46.4%)
	Female	180 (53.6%)
**Resident status**		
	Urban	184 (54.8%)
	Rural	152 (45.2%)
**Education**		
	Junior middle school	31 (9.2%)
	High school	96 (28.6%)
	Junior college	68 (20.2%)
	Bachelor’s degree	127 (37.8%)
	Master’s degree	9 (2.7%)
	Doctor’s degree	5 (1.5%)
**Job**		
	Private business owners	28 (8.3%)
	Factory workers	31 (9.2%)
	Professional and technical workers	77 (22.9%)
	Commercial service workers	63 (18.8%)
	Students	38 (11.3%)
	Liberal professionals	27 (8.0%)
	Employees in government offices and public institutions	40 (11.9%)
	Retirees	22 (6.5%)
	Farmers	10 (3.0%)

## Results

### Data Analysis

We analyzed data on the basis of methods from previous studies [1,56]. The reliability and validity of the measures were analyzed using the SPSS 22.0 software. Cronbach alpha, which was used to assess the reliability, needed to be at least .700 [59]. Table 2 presents the Cronbach alpha of each construct, and these results show that the scale in this study had good reliability. The Kaiser-Meyer-Olkin value (weak, .500; medium, .600; good, .700; very good, .800; and perfect, .900) [65-68] was equal to .907 (*P*<.001, significant) above the cutoff value of .900; thus, the construct validity was fully acceptable.

In accordance with a study by Wu et al [69], we evaluated the discriminant validities of the constructs and ensured whether internet health information quality, the source of internet health information, CBT and ABT, and patient compliance were distinct from each other and from the indicators loaded onto their intended latent variables, by means of nested confirmatory factor analytic models. We established and compared 6 nested models based on the research model (see Figure 1): (1) a 5-factor model treating each of the variables as separate factors; (2) a 4-factor model treating internet health information quality and the source of internet health information as the first factor, CBT as the second factor, ABT as the third factor, and patient compliance as the fourth factor; (3) a 4-factor model treating internet health information quality as the first factor, treating the source of internet health information as the second factor, CBT and ABT as the third factor, and patient compliance as the fourth factor; (4) a 3-factor model treating internet health information quality and source of internet health information as the first factor, CBT and ABT as the second factor, and patient compliance as the third factor; (5) a 2-factor model treating internet health information quality, the source of internet health information, CBT, and ABT as the first factor and patient compliance as the second factor; and (6) a 1-factor model treating all 5 factors as one factor. As shown in Table 3, there was a good fit between the data and the 5-factor model (model 1; χ^2^_1390_=1793.1, χ^2^/*df*=1.29<3; comparative fit index=.96>.90; Tucker-Lewis index=.95>.90; incremental fit index=.96>.90; root mean square error of approximation=.029<.050) [70-74]. Compared with model 1, the other 5 nested models (Models 2-6) were worse fits to the data, according to all fit indices. Therefore, we concluded that internet health information quality, the source of internet health information, CBT and ABT, and patient compliance were 5 different factors.

### Hypothesis Testing

The demographical statistics were used to identify any significant relationship between the demographic factors and variables of the research model [1]. Our analytical results indicated the following: (1) Gender: the relationship between gender and CBT and that between gender and patient compliance was significant. Specifically, females were more likely than males to cognitively trust their physicians and behaved with higher compliance. (2) Age: according to the results of the analysis of covariance, age exhibited a marked effect on the relationship between internet health information quality and patient compliance and between ABT and patient compliance. (3) Job: private business owners held more positive attitudes about the sources of internet health information (health websites) than commercial service workers, students, and liberal professionals. Therefore, we added gender, age, and job as control variables into the research model.

**Table 2:** Cronbach alpha of the constructs.

Constructs	Cronbach alpha
Internet health information quality	.933
Source of internet health information	.910
Cognition-based trust (CBT)	.865
Affect-based trust (ABT)	.756
Patient compliance	.870
Total^a^	.950

^a^For the total value, all five constructs were regarded as one and were used to calculate the total Cronbach alpha.

**Table 3:** Comparison of measurement models in confirmatory factor analysis.

Distinctiveness test for all variables (model factors)	Fit indices					
	χ^2a^ (*df*^b^)	χ^2^/*df*	RMSEA^c^	CFI^d^	IFI^e^	TLI^f^
Model 1 (5 factors): internet health information quality, source of internet health information, CBT^g^, ABT^h^, patient compliance	1793.1 (1390)	1.29	.029	.96	.96	.95
Model 2 (4 factors): internet health information quality and source of internet health information combined into 1 factor	2322.5 (1394)	1.67	.045	.91	.92	.89
Model 3 (4 factors): CBT and ABT combined into 1 factor	1878.8 (1394)	1.35	.032	.96	.96	.94
Model 4 (3 factors): internet health information quality and source of internet health information combined into 1 factor and CBT and ABT combined into 1 factor	2399.4 (1397)	1.72	.046	.91	.91	.88
Model 5 (2 factors): internet health information quality, source of internet health information, CBT and ABT combined into 1 factor	3046.8 (1399)	2.18	.059	.85	.85	.81
Model 6 (1 factor): internet health information quality, source of internet health information, CBT and ABT, and patient compliance combined into 1 factor	3303.1 (1400)	2.36	.064	.83	.83	.78

^a^χ^2^: Pearson chi-square.

^b^*df*: degrees of freedom.

^c^RMSEA: root mean square error of approximation.

^d^CFI: comparative fit index.

^e^IFI: incremental fit index.

^f^TLI: Tucker-Lewis index.

^g^CBT: cognition-based trust.

^h^ABT: affect-based trust.

First, we used a hierarchical multiple linear regression method to test our hypotheses and to evaluate the effects of the control variables. Table 4 presents the path coefficient and significance of each relationship and shows that the relationships proposed by H1, H2, H3, H5, H6, and H7 were significant; however, the relationship hypothesized by H4 was nonsignificant. In addition, Cohen ƒ^2^ [75] was used to assess the effects of the control variables, with results divided into several categories (ie, insignificant: <.020; small: ≥.020 and <.150; medium: ≥.150 and <.300; and large: ≥.350). As shown in Table 5, the effect sizes of gender, age, and job were all small. Table 6 shows the effect sizes of variables.

We found that only the effect size of CBT on ABT was large, whereas the effect size of CBT on patient compliance was small. In addition, the effect sizes of the quality as well as source of internet health information and ABT were all small.

**Table 4:** Results of hierarchical multiple linear regression.

Hypothesis	Path coefficient	*P* value
H1: Internet health information quality → CBT^a^	.317	<.001
H2: Internet health information quality → ABT^b^	.213	<.001
H3: Source of internet health information → CBT	.224	<.001
H4: Source of internet health information → ABT	.076	.13
H5: CBT → patient compliance	.326	<.001
H6: ABT → patient compliance	.378	<.001
H7: CBT → ABT	.535	<.001

^a^CBT: cognition-based trust.

^b^ABT: affect-based trust.

**Table 5:** Multivariate coefficient of determination (*R*^2^) results, where *∆R*^2^ is *R*^2^_with control variables_ − *R*^2^_without control variables_.

Variables	*R*^2^		Control variable effects		
	With control variables	Without control variables	∆*R*^2^	Cohen ƒ^2^	Effects
Cognition-based trust (CBT)	0.252	0.229	0.023	0.031	Small
Affect-based trust (ABT)	0.487	0.470	0.017	0.033	Small
Patient compliance	0.461	0.443	0.018	0.033	Small

**Table 6:** Hierarchical multiple linear regression effect size analysis, where *R*^2^ is multivariate coefficient of determination and *∆R*^2^ is *R*^2^_with control variables_ − *R*^2^_without control variables_.

Variables		*R*^2^		∆*R*^2^	Cohen ƒ^2^	Effect size
		In	Out			
**Patient compliance**						
	Cognition-based trust (CBT)	.461	.406	.055	.102	Small
	Affect-based trust (ABT)	.461	.388	.073	.135	Small
**Cognition-based trust (CBT)**						
	Internet health information quality	.252	.185	.067	.090	Small
	Source of internet health information	.252	.219	.033	.044	Small
**Affect-based trust (ABT)**						
	Internet health information quality	.487	.459	.028	.055	Small
	Source of internet health information	.487	.483	.004	.008	Small
	Cognition-based trust (CBT)	.487	.272	.215	.419	Large

**Figure 2:**
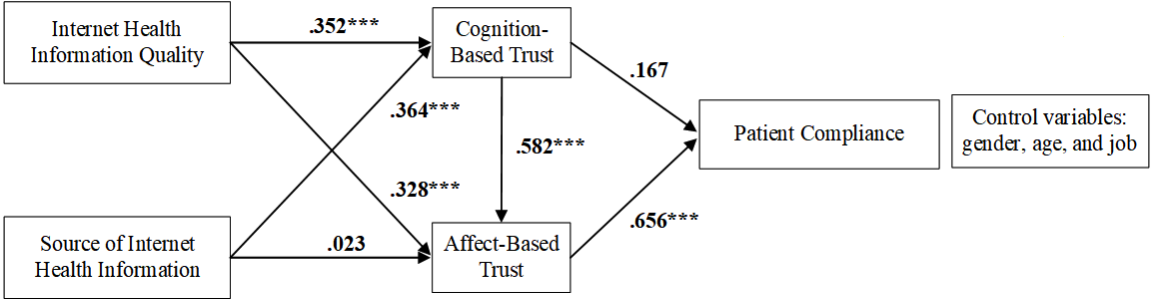
Research model with path coefficients. ***P<.001, *P<.05.

We used SPSS 22.0 and Amos 22.0 to test our hypotheses and found that age had a positive effect on patient compliance and that older patients were more likely to follow their physicians’ suggestions. Females were more willing to cognitively trust their physicians and comply with their physicians, whereas males were more likely to have ABT in their physicians. The job type only significantly impacted ABT. Specifically, liberal professions held the highest ABT in their physicians, whereas private business owners were least likely to affectively trust their physicians.

Figure 2 indicates the SEM results, and the magnitude and significance of the path coefficients are shown in Table 7. Five hypotheses were supported (H1, H2, H3, H6, and H7), but H4 and H5 were not supported, and we have provided the possible reasons for these nonsignificant relationships in the next section. In addition, we used the bootstrapping method (n=5000, 95% CI) to further analyze the mediating effects in the research model.

As Table 8 reveals, we first concluded that the significant mediating effects of CBT and ABT between the source of internet health information and patient compliance were nonsignificant. In addition, Sobel test was used to evaluate the mediating role played by CBT between internet health information quality and patient compliance. To specify, we found that the value of *Z* was .489, significantly lower than .900, indicating that the mediation of CBT was nonsignificant. Furthermore, ABT had a significant mediating effect on the relationship between internet health information quality and patient compliance, with all the effects (direct, indirect, and total) being significant.

**Table 7:** Hypothesis testing.

Hypothesis	Path coefficient	*P* value
Internet health information quality has a positive impact on patients’ CBT^a^ in their physicians.	.352	<.001
Internet health information quality has a positive impact on patients’ ABT^b^ in their physicians.	.328	<.001
The source of the internet health information has a positive impact on patients’ CBT in their physicians.	.364	<.001
The source of the internet health information has a positive impact on patients’ ABT in their physician.	.023	.72
Patients’ CBT in their physicians has a positive impact on the compliance.	.167	.09
Patients’ ABT in their physicians has a positive impact on the compliance.	.656	<.001
Patients’ CBT in their physicians has a positive impact on their ABT in physicians.	.582	<.001

^a^CBT: cognition-based trust.

^b^ABT: affect-based trust.

**Table 8:** Path coefficients by bootstrapping method. Amos 22.0 used to calculate direct, indirect, and total effects.

Effect		Path coefficient (SD)	*P* value
**Direct effects**			
	internet health information quality → CBT^a^	.309 (.076)	.001
	source of internet health information → CBT	.310 (.069)	<.001
	CBT → patient compliance	.142 (.288)	.46
	internet health information quality → ABT^b^	.308 (.073)	<.001
	source of internet health information → ABT	.062 (.074)	.39
	ABT → patient compliance	.737 (.330)	.001
**Indirect effects**			
	internet health information quality → patient compliance	.412 (.111)	<.001
	source of internet health information → patient compliance	.232 (.081)	.008
**Total effects**			
	internet health information quality → patient compliance	.393 (.067)	<.001
	source of internet health information → patient compliance	.083 (.076)	.28

^a^CBT: cognition-based trust.

^b^ABT: affect-based trust.

## Discussion

### Principal Results

This is the first study that discusses patient compliance from the perspective of both CBT and ABT. This study makes several theoretical contributions and practical implications for future study of patient compliance and ways to improve the patient-physician relationship. First, we constructed a research model to identify the relationship between internet health information and patient compliance, mediated by trust. We clarified the mechanisms through which internet health information (quality and source) impacts patient compliance. To specify, we used CBT and ABT that patients have in their physicians as the mediators. Internet health information quality directly impacts patients’ CBT and ABT in their physicians and indirectly impacts patient compliance. Laugesen et al [1] identified the indirect impact that high-quality internet health information could increase patient compliance. Therefore, patient compliance can be improved by strengthening the management of certain aspects of internet health information quality, such as the information topics, categories, meaning, and usability. In addition, the path coefficient from internet health information quality to CBT (.352) was higher than that from internet health information quality to ABT (0.328), and the source of internet health information only directly affected CBT but did not have any direct effect on ABT. Hence, we found that internet health information (quality and source) had more significant impacts on CBT than on ABT, implying that individuals always rationally deal with internet health information on the basis of their own cognition. Thus, CBT in the patient-physician relationship can be improved by improving the quality and source of internet health information. For example, physicians should focus on the health information obtained from health websites to understand the health-information-seeking preferences of their patients. Moreover, physicians should communicate with their patients using health websites, which can enable them to share health information with their patients through Web and establish CBT.

Second, ABT has positive effects on patient compliance, but we did not identify any significant direct effect of CBT on patient compliance. In accord with Mcallister’s [34] proposal, we too found that CBT directly impacts ABT, thus, indirectly impacting patient compliance. In other words, ABT has a mediating effect on the relationship between CBT and patient compliance. Lee et al [76] reported that trust in physicians was related to patient compliance, such that when patients highly trusted their physicians, they appeared to be more likely to report their health status to these physicians; this finding indicates that patient compliance can be improved by enhancing patients’ CBT and ABT in their physicians. On the one hand, physicians are advised to enrich their knowledge structures to show their specialization and reliability in the interaction with their patients to inspire their CBT. On the other hand, communication is important in the patient-physician relationship, and physicians should focus on mutual care and respect for patients in communication to promote patients to affectively trust their physicians. In addition, we suggest that physicians obtain internet health information to enrich themselves and they should actively participate in discussions with their patients on health websites to establish a good atmosphere for communication.

Third, and surprisingly, a nonsignificant relationship was found between the source of internet health information and ABT in physicians. We considered that there might be suppression effects of other factors in the model. Thus, we removed the relationships between (1) internet health information quality and ABT, (2) the source of internet health information and CBT, and (3) CBT and ABT and retained only the relationship between the source of internet health information and ABT. Then, we added the three abovementioned relationships to the model one by one and investigated how each of them affected the strength of the path from the source of internet health information to ABT. Eventually, we found that the quality of internet health information played a critical role in impacting the relationship between the source of internet health information and ABT. Furthermore, this relationship became nonsignificant when the relationship between internet health information quality and ABT was added to the model. According to the results of bootstrapping, both the quality and the source of internet health information have indirect effects on patient compliance, but only the quality totally affects patient compliance. In addition, the path coefficient of the indirect relationship between internet health information quality and patient compliance is larger than that between internet health information source and compliance. In fact, ABT is based on an emotional connection and subjective judgment. Therefore, the quality of internet health information may be stronger than the source, from the patients’ point of view, because the demand for quality may be greater than that for the source whose impact on ABT was ignored in this analysis.

### Limitations

The limitations of the study must be considered. First, we focused on only 2 dimensions of internet health information: quality and source. Other dimensions may also be worthy of investigation. Second, in this study, we examined the relationship between internet health information and patient compliance in the context of China. However, China is a special country in terms of health care due to its large population and unbalanced health care development in different areas. In addition, China is currently actively promoting Web-based health care. There may be several common and different aspects between China and other places, which can be addressed in future research through additional surveys. Third, a cross-sectional survey was used to collect data from respondents. Thus, changes in patient compliance that were associated with the attitudes of patients toward internet health information may not have been captured. Fourth, this study proposed universal and guiding suggestions on the basis of discussions about internet health information quality and source. Future studies may aim to evaluate the quality and source of internet health information, focusing on and collecting user data from specific health websites. Fifth, all concepts and relationships were measured only once. This study was conducted from a static perspective and, therefore, failed to consider dynamic changes in patient attitudes. Last, although our sample met the characteristics of typical internet health information seekers, we did not consider the feature of Chinese census data, and the number of respondents was relatively small.

### Conclusions

This study indicates that both the quality and source of internet health information markedly impact patient compliance through the mediations of CBT and ABT. In our research model, the quality of internet health information showed a stronger effect on patient compliance than the source of that information and the information quality also showed a positive impact on CBT and ABT. Consequently, health information quality indirectly affects patient compliance. In terms of trust, ABT appears to have a stronger effect than CBT on patient compliance. These findings suggest the follwing: (1) patient compliance can be improved by strengthening the management of certain aspects of internet health information quality, such as its topics, categories, meaning, and usability; (2) physicians could focus on obtaining information from health websites to understand patients’ health-information-seeking preferences and to enrich their own knowledge to enhance their specialization and reliability; (3) physicians can communicate with patients on health websites and share information as well as establish CBT with them through Web; and (4) a mutual demonstration of care and respect in the communication between physicians and patients is important during treatment and is beneficial in promoting patients’ affective trust in their physicians.

## Appendix

Multimedia Appendix 1 Measurement instruments.

## Abbreviations

ABT: affect-based trust
CBT: cognition-based trust
SEM: structural equation modeling

## References

[1] Laugesen J, Hassanein K, Yuan Y (2015). The Impact of Internet Health Information on Patient Compliance: A Research Model and an Empirical Study. J Med Internet Res.

[2] Tustin N (2010). The role of patient satisfaction in online health information seeking. J Health Commun.

[3] Deng Z, Liu S, Hinz O (2015). The health information seeking and usage behavior intention of Chinese consumers through mobile phones. Info Technology & People.

[4] Horwitz RI, Horwitz SM (1993). Adherence to treatment and health outcomes. Arch Intern Med.

[5] Kassirer JP (2000). Patients, physicians, and the Internet. Health Aff (Millwood).

[6] Ruppel EK (2016). Scanning Health Information Sources: Applying and Extending the Comprehensive Model of Information Seeking. J Health Commun.

[7] Hu Y, Shyam Sundar S (2009). Effects of Online Health Sources on Credibility and Behavioral Intentions. Communication Research.

[8] Cohn A, Richters J (2013). ‘My Vagina Makes Funny Noises’: Analyzing Online Forums to Assess the Real Sexual Health Concerns of Young People. International Journal of Sexual Health.

[9] Mendes Á, Abreu L, Vilar-Correia MR, Borlido-Santos J (2017). “That Should Be Left to Doctors, That's What They are There For!”-Exploring the Reflexivity and Trust of Young Adults When Seeking Health Information. Health Commun.

[10] Clarke MA, Moore JL, Steege LM, Koopman RJ, Belden JL, Canfield SM, Meadows SE, Elliott SG, Kim MS (2016). Health information needs, sources, and barriers of primary care patients to achieve patient-centered care: A literature review. Health Informatics J.

[11] Yang Q, Chen Y, Wendorf MJ (2017). Social Support, Trust in Health Information, and Health Information-Seeking Behaviors (HISBs): A Study Using the 2012 Annenberg National Health Communication Survey (ANHCS). Health Commun.

[12] Seçkin G, Yeatts D, Hughes S, Hudson C, Bell V (2016). Being an Informed Consumer of Health Information and Assessment of Electronic Health Literacy in a National Sample of Internet Users: Validity and Reliability of the e-HLS Instrument. J Med Internet Res.

[13] Salancik GR, Pfeffer J (1978). A social information processing approach to job attitudes and task design. Adm Sci Q.

[14] Doi Suhail A R, Amigo Maria Florencia (2007). Nurses' intentions to wear gloves during venipuncture procedures: a behavioral psychology perspective. Infect Control Hosp Epidemiol.

[15] Swindell JS, McGuire AL, Halpern SD (2010). Beneficent persuasion: techniques and ethical guidelines to improve patients' decisions. Ann Fam Med.

[16] Umaki TM, Umaki MR, Cobb CM (2012). The psychology of patient compliance: a focused review of the literature. J Periodontol.

[17] Ritterband LM, Borowitz S, Cox DJ, Kovatchev B, Walker LS, Lucas V, Sutphen J (2005). Using the internet to provide information prescriptions. Pediatrics.

[18] Li F, Li M, Guan P, Ma S, Cui L (2015). Mapping publication trends and identifying hot spots of research on Internet health information seeking behavior: a quantitative and co-word biclustering analysis. J Med Internet Res.

[19] Pang PC, Chang S, Verspoor K, Pearce J (2016). Designing Health Websites Based on Users' Web-Based Information-Seeking Behaviors: A Mixed-Method Observational Study. J Med Internet Res.

[20] Maloney EK, D'Agostino TA, Heerdt A, Dickler M, Li Y, Ostroff JS, Bylund CL (2015). Sources and types of online information that breast cancer patients read and discuss with their doctors. Palliat Support Care.

[21] Rowley J, Johnson F, Sbaffi L (2015). Gender as an influencer of online health information-seeking and evaluation behavior. J Assn Inf Sci Tec.

[22] Kostagiolas P, Korfiatis N, Kourouthanasis P, Alexias G (2014). Work-related factors influencing doctors search behaviors and trust toward medical information resources. International Journal of Information Management.

[23] Sbaffi L, Rowley J (2017). Trust and Credibility in Web-Based Health Information: A Review and Agenda for Future Research. J Med Internet Res.

[24] Provost M, Koompalum D, Dong D, Martin BC (2006). The initial development of the WebMedQual scale: domain assessment of the construct of quality of health web sites. Int J Med Inform.

[25] Khera AV, Emdin CA, Drake I, Natarajan P, Bick AG, Cook NR, Chasman DI, Baber U, Mehran R, Rader DJ, Fuster V, Boerwinkle E, Melander O, Orho-Melander M, Ridker PM, Kathiresan S (2016). Genetic Risk, Adherence to a Healthy Lifestyle, and Coronary Disease. N Engl J Med.

[26] Stonerock GL, Blumenthal JA (2017). Role of Counseling to Promote Adherence in Healthy Lifestyle Medicine: Strategies to Improve Exercise Adherence and Enhance Physical Activity. Prog Cardiovasc Dis.

[27] Johal S, Jamsen KM, Bell JS, Mc NKP, Magliano DJ, Liew D, Ryan-Atwood TE, Anderson C, Ilomäki J (2017). Do statin users adhere to a healthy diet and lifestyle? The Australian Diabetes, Obesity and Lifestyle Study. Eur J Prev Cardiol.

[28] Grosso G, Bella F, Godos J, Sciacca S, Del RD, Ray S, Galvano F, Giovannucci EL (2017). Possible role of diet in cancer: systematic review and multiple meta-analyses of dietary patterns, lifestyle factors, and cancer risk. Nutr Rev.

[29] Stacey FG, Lubans DR, Chapman K, Bisquera A, James EL (2017). Maintenance of Lifestyle Changes at 12-month Follow-up in a Nutrition and Physical Activity Trial for Cancer Survivors. Am J Health Behav.

[30] Varleta P, Acevedo M, Akel C, Salinas C, Navarrete C, García A, Echegoyen C, Rodriguez D, Gramusset L, Leon S, Cofré P, Retamal R, Romero K (2017). Mobile phone text messaging improves antihypertensive drug adherence in the community. J Clin Hypertens (Greenwich).

[31] Hensher DA, Rose JM, Greene WH (2015). Applied Choice Analysis.

[32] Brown MT, Bussell J, Dutta S, Davis K, Strong S, Mathew S (2016). Medication Adherence: Truth and Consequences. Am J Med Sci.

[33] Zhao D, Rao K, Zhang Z (2016). Patient Trust in Physicians: Empirical Evidence from Shanghai, China. Chin Med J (Engl).

[34] McAllister DJ (1995). AFFECT- AND COGNITION-BASED TRUST AS FOUNDATIONS FOR INTERPERSONAL COOPERATION IN ORGANIZATIONS. Academy of Management Journal.

[35] Casimir G, Lee K, Loon M (2012). Knowledge sharing: influences of trust, commitment and cost. J of Knowledge Management.

[36] Choi BK, Moon HK, Nae EY (2014). Cognition- and affect-based trust and feedback-seeking behavior: the roles of value, cost, and goal orientations. J Psychol.

[37] Massey GR, Dawes PL (2007). Personal characteristics, trust, conflict, and effectiveness in marketing/sales working relationships. European Journal of Marketing.

[38] Parayitam S, Dooley RS (2009). The interplay between cognitive- and affective conflict and cognition- and affect-based trust in influencing decision outcomes. Journal of Business Research.

[39] Wang J, Zhang Z, Jia M (2016). Understanding How Leader Humility Enhances Employee Creativity. The Journal of Applied Behavioral Science.

[40] Barker RT, Camarata MR (1998). The Role of Communication in Creating and Maintaining a Learning Organization: Preconditions, Indicators, and Disciplines. Journal of Business Communication.

[41] Schwaer C, Biemann T, Voelpel S (2012). Antecedents of employee's preference for knowledge-sharing tools. The International Journal of Human Resource Management.

[42] Kim Y (2016). Trust in health information websites: A systematic literature review on the antecedents of trust. Health Informatics J.

[43] Hsiung HH, Tsai WC (2017). The Joint Moderating Effects of Activated Negative Moods and Group Voice Climate on the Relationship between Power Distance Orientation and Employee Voice Behavior. Applied Psychology.

[44] Young GJ, Meterko MM, Mohr D, Shwartz M, Lin H (2009). Congruence in the assessment of service quality between employees and customers: A study of a public health care delivery system. Journal of Business Research.

[45] Boekhorst JA (2014). The Role of Authentic Leadership in Fostering Workplace Inclusion: A Social Information Processing Perspective. Hum Resour Manage.

[46] Blau PM (1964). Exchange and Power in Social Life.

[47] Foa UG, Foa EB (1974). Societal structures of the mind.

[48] Foa EB, Foa UG (1976). Resource theory of social exchange. Contemporary Topics in Social Psychology.

[49] Barbouni A, Nalmpanti M, Gennimata D, Theodoridis D, Merakou K (2017). Beliefs and practices of Greek doctors in relation to patients' adherence to antihypertensive medication. J Hum Hypertens.

[50] Pappas JM, Flaherty KE (2008). The effect of trust on customer contact personnel strategic behavior and sales performance in a service environment. Journal of Business Research.

[51] Moorman C, Zaltman G, Deshpande R (1992). Relationships between Providers and Users of Market Research: The Dynamics of Trust within and between Organizations. Journal of Marketing Research.

[52] Ha H, John J, John JD, Chung Y (2016). Temporal effects of information from social networks on online behavior. Internet Research.

[53] Rutten W, Blaas-Franken J, Martin H (2016). The impact of (low) trust on knowledge sharing. J of Knowledge Management.

[54] Emerson RM (1981). Social exchange theory. Social Psychology: Sociological Perspectives.

[55] Singh AG, Singh S, Singh PP (2012). YouTube for information on rheumatoid arthritis--a wakeup call?. J Rheumatol.

[56] Wiertz C, de Ruyter K (2016). Beyond the Call of Duty: Why Customers Contribute to Firm-hosted Commercial Online Communities. Organization Studies.

[57] Hoque R, Sorwar G (2017). Understanding factors influencing the adoption of mHealth by the elderly: An extension of the UTAUT model. Int J Med Inform.

[58] Gefen D, Straub DW, Boudreau MC (2000). Structural equation modeling and regression: Guidelines for research practice. Communications of the Association for Information Systems.

[59] Buysse HEC, Coorevits P, Van MG, Hutse A, Kaufman J, Ruige J, De MGJE (2010). Introducing telemonitoring for diabetic patients: development of a telemonitoring 'Health Effect and Readiness' Questionnaire. Int J Med Inform.

[60] Sardeshmukh S, Vandenberg RJ (2013). Integrating moderation and mediation: a structural equation modeling approach. AMPROC.

[61] Bu X, You L, Li Y, Liu K, Zheng J, Yan T, Chen S, Zhang L (2017). Psychometric Properties of the Kessler 10 Scale in Chinese Parents of Children With Cancer. Cancer Nurs.

[62] Xiao Y, Li T, Xiao L, Wang S, Wang S, Wang H, Wang B, Gao Y (2017). The Chinese version of Instrument of Professional Attitude for Student Nurses (IPASN): Assessment of reliability and validity. Nurse Educ Today.

[63] Wong WS, Chen PP, Chow YF, Wong S, Fielding R (2016). A Study of the Reliability and Concurrent Validity of the Chinese Version of the Pain Medication Attitude Questionnaire (ChPMAQ) in a Sample of Chinese Patients with Chronic Pain. Pain Med.

[64] Wimble M (2016). Understanding Health and Health-Related Behavior of Users of Internet Health Information. Telemed J E Health.

[65] Kaiser HF (1970). A second generation little jiffy. Psychometrika.

[66] Kurtuldu MK, Bulut D (2017). Development of a Self-Efficacy Scale toward Piano Lessons. Educational Sciences: Theory and Practice.

[67] Biasutti M, Frate S (2016). A validity and reliability study of the Attitudes toward Sustainable Development scale. Environmental Education Research.

[68] Erdogan M, Ok A, Marcinkowski TJ (2012). Development and validation of Children’s Responsible Environmental Behavior Scale. Environmental Education Research.

[69] Wu W, Tang F, Dong X, Liu C (2014). Different identifications cause different types of voice: A role identity approach to the relations between organizational socialization and voice. Asia Pac J Manag.

[70] Nuño-Solinís R, Berraondo ZI, Sauto AR, San MRL, Toro PN (2013). Development of a questionnaire to assess interprofessional collaboration between two different care levels. Int J Integr Care.

[71] Mu GM, Hu Y (2016). Validation of the Chinese Version of the 12-Item Child and Youth Resilience Measure. Children and Youth Services Review.

[72] Bentler PM, Bonett DG (1980). Significance tests and goodness of fit in the analysis of covariance structures. Psychological Bulletin.

[73] Steiger JH, Lind J (1980). Statistically based tests for the number of common factors.

[74] Bentler PM, Kano Y (1990). On the Equivalence of Factors and Components. Multivariate Behav Res.

[75] Cohen J (1988). Statistical power analysis for the behavioral sciences. Journal of the American Statistical Association.

[76] Lee Y, Lin JL (2009). The effects of trust in physician on self-efficacy, adherence and diabetes outcomes. Soc Sci Med.

