# Supplementary material for: Relationship Between Internet Health Information and Patient Compliance Based on Trust: Empirical Study
Source: J Med Internet Res. 2018 Aug 17;20(8):e253. doi: 10.2196/jmir.9364 (PMC6119214; doi:10.2196/jmir.9364)
Supplement: Multimedia Appendix 1 [file jmir_v20i8e253_app1.pdf]

Multimedia Appendix 1

Table A1. Measurement instruments.

Construct	Scale or Scoring	Items
Internet health information quality [1]	• 7-point Likert-type scale<br>• Strongly disagree to strongly agree	**Relevance:**
		1. For your health information needs, to what degree do you believe the internet health information provided by the website was applicable to your needs?
		2. For your health information needs, to what degree do you believe internet health information provided by the website was related to your needs?
		3. For your health information needs, to what degree do you believe internet health information provided by the website was pertinent to your needs?
		4. For your health information needs, to what degree do you believe internet health information provided by the website was relevant to your needs?
		**Understandability:**
		1. For your health information needs, to what degree do you believe internet health information provided by the website was clear in meaning?
		2. For your health information needs, to what degree do you believe internet health information provided by the website was easy to read?
		3. For your health information needs, to what degree do you believe internet health information provided by the website was easy to comprehend?
		4. For your health information needs, to what degree do you believe internet health information provided by the website was understandable?
		**Adequacy:**
		1. For your health information needs, to what degree do you believe internet health information provided by the website was sufficient?

		2. For your health information needs, to what degree do you believe internet health information provided by the website was complete?
		3. For your health information needs, to what degree do you believe internet health information provided by the website was adequate?
		4. For your health information needs, to what degree do you believe internet health information provided by the website contained the necessary topics or categories?
		**Usefulness:**
		1. For your health information needs, to what degree do you believe internet health information provided by the website was informative?
		2. For your health information needs, to what degree do you believe internet health information provided by the website was valuable?
		3. For your health information needs, to what degree do you believe internet health information provided by the website was helpful?
		4. For your health information needs, to what degree do you believe internet health information provided by the website was useful?
Source of internet health information [24,55]	● 7-point Likert-type scale<br>● Strongly disagree to strongly agree	**Reliability** [55]
		1. Are the aims clear and achieved?
		2. Are reliable sources of internet health information used? (ie, publication cited, speaker is board-certified rheumatologist)
		3. Is the internet health information presented balanced and unbiased?
		4. Are additional sources of internet health information listed for patient reference?
		5. Are areas of uncertainty mentioned?
		**Authority** [24]
		1. Is the source of internet health information author identified?
		2. Did medically trained and qualified professionals develop the internet health information?

		3. Is the author(s)' occupation, experience, training and education clearly stated?
		4. Is there an editorial board or a listing of the names of those responsible for preparing and reviewing the content of the internet health information's source?
		5. Are the credentials of those responsible for preparing and reviewing the content of the internet health information's source mentioned?
		6. If the source of internet health information provides a discussion board to its users, does it state that the comments appearing on the discussion board are only opinions of the individuals using the discussion board?
		7. Is copyright ownership of specific content clearly indicated?
		8. Are the sources of internet health information ownership(s) and commercial interests, including affiliations, strategic alliances, and significant investors, clearly indicated on or linked from the home page?
		9. As the source of internet health information, are affiliations and financial disclosures relevant to authors and content producers clearly indicated?
		10. As the source of internet health information, is funding or other sponsorship for any specific content clearly indicated?
		11. As the source of internet health information, is there a link to the page describing the purpose of the sponsoring organization?
		12. As the source of internet health information, does acknowledgment of support appear on the home page, the running foot of all pages, and on any materials used to publicize the Web-based product?
		13. As the source of internet health information, does the site encourage individuals who post content in Web-based discussions, chat rooms, and e-lists to disclose financial interests and commercial funding or affiliations related to the subject of the posted content discussion, chat, or list?

		14. On the source of internet health information, is the content easily distinguished from advertising?
		15. On the source of internet health information, does the advertising detract from the credibility of the information?
		16. On the source of internet health information, if advertising is a source of funding, is it clearly stated?
		17. As the source of internet health information, if the site has advertising, does the site state the advertising policy?
		18. On the source of internet health information, is the advertising adjacent to (ie, next to or within) the editorial content on the same topic?
		**Accessibility** [24]
		1. Does the source of internet health information provide a choice of more than one language?
		2. Do you need to register to use the source of internet health information?
		3. Is the source of internet health information free that you always use?
		4. Are portions of the source of internet health information unavailable because they are "under construction"?
		5. Does the source of internet health information accommodate users with disabilities?
Cognition-based trust [34]	● 7-point Likert-type scale<br>● Strongly disagree to strongly agree	1. This physician approaches his or her job with professionalism and dedication.
		2. Given this physician's track record, I see no reason to doubt his or her competence and preparation for the job.
		3. I can rely on this physician not to make my treatment more difficult by careless work.
		4. Most people, even those who are not close friends of this individual, trust and respect him or her.
		5. Other relatives and friends of mine who must interact with this physician consider him or her to be trustworthy.

		6. If people knew more about this physician and his or her background, would they be more concerned and monitor his or her performance more closely?
Affect-based trust [34]	● 7-point Likert-type scale<br>● Strongly disagree to strongly agree	1. I have a sharing relationship with physicians. We can both freely share our ideas, feelings, and hopes.
		2. I can talk freely to this individual about difficulties I am having in treatment and know that (s)he will want to listen.
		3. I would feel a sense of loss if my physician was transferred and I could no longer be treated by her or him.
		4. If I shared my problems with this physician, I know (s)he would respond constructively and caringly.
		5. I would have to say that we have both made considerable emotional investments in our patient-physician relationship.
Patient compliance [1]	● 7-point Likert-type scale<br>● Strongly disagree to strongly agree	1. I am following or did follow the physician’s suggestions of treatment exactly.
		2. I am following or did follow the physician’s recommendations of drug or medication with efficacy.
		3. I am following or did follow the physician’s orders for treatment, such as to stay in bed.
		4. I have returned or plan to return to the physician on the schedule he or she suggested for treatment.
		5. I have had or plan to have the follow-up tests for treatment, as recommended by the physician.
